# Case Report: Detection of Double ROS1 Translocations, *SDC4-ROS1* and *ROS1-GK*, in a Lung Adenocarcinoma Patient and Response to Crizotinib

**DOI:** 10.3389/fmed.2021.649177

**Published:** 2021-09-20

**Authors:** Long Xu, Xiaoxia Chen, Hong Huo, Yongye Liu, Xiaodan Yang, Dejian Gu, Mingming Yuan, Min Zhang, Rongrong Chen, Jiayin Wang, Zhendong Zheng

**Affiliations:** ^1^Department of Oncology, General Hospital of Northern Theater Command, Shenyang, China; ^2^Department of Nephrology, The Chinese People's Liberation Army Rocket Force Characteristic Medical Center, Beijing, China; ^3^Geneplus-Beijing, Beijing, China; ^4^School of Computer Science and Technology, Xi'an Jiaotong University, Xi'an, China

**Keywords:** next-generation sequencing, lung adenocarcinoma, double *ROS1* fusion, crizotinib, novel *ROS1* fusion

## Abstract

*ROS1* rearrangement, identified in ~2% of non-small cell lung cancer (NSCLC), has defined a distinctive molecular subtype. Patients with *ROS1* fusion have been shown to be highly sensitive to treatment with crizotinib. However, the efficacy of crizotinib in NSCLC patients with double *ROS1* fusions remains to be elucidated. Here, we report a 40-year-old male diagnosed with stage IIIA lung adenocarcinoma. Two *ROS1* fusions [SDC4-*ROS1* (EX2:EX32) and *ROS1*-GK (EX31:EX13)] were detected simultaneously in tumor tissue of this patient by next-generation sequencing. Crizotinib was administered, and the patient showed a partial response in lung lesions. Nevertheless, a brain lesion was found at 8 months after treatment. The slightly short duration of response may be related to the presence of *ROS1-GK* rearrangement. This case proved that patients with *SDC4-ROS1* and *ROS1-GK* fusions may be sensitive to crizotinib, but short progression-free survival of this case showed that the presence of ROS1-GK rearrangement may affect the efficacy of crizotinib. A large-scale investigation on the efficacy of *ROS1* inhibitors in patients with complex *ROS1* fusions should be conducted in the future.

## Introduction

Lung cancer is the leading cause of cancer-related mortality worldwide, and non-small cell lung cancer (NSCLC) represents the major histological subtype of the disease (1). The ROS proto-oncogene 1, receptor tyrosine kinase (*ROS1*) gene has been proven to be a valuable therapeutic target in NSCLC patients. The prevalence of *ROS1* rearrangements is estimated to be 1–2% in NSCLC patients (2). *CD74* is the most common *ROS1* fusion partner. To date, at least 20 fusion partners have been reported in NSCLC, including *SLC34A2, ADGRG6*, and *GOPC* (3–5). With the rapid development of gene sequencing technologies, more and more *ROS1* fusion partners are being identified, with their responses to crizotinib reported in several case reports (6, 7).

Crizotinib is an anaplastic lymphoma kinase (*ALK*)/*ROS1*/MET inhibitor, and it has become the first targeted agent approved by the US Food and Drug Administration for the treatment of advanced *ROS1*-rearranged NSCLC (8, 9). Despite dramatic response to crizotinib therapy in patients with *ROS1* rearrangements, the response duration varies among patients with different clinical and genetic characteristics. Compared to patients with *CD74-ROS1* fusion, patients with non-*CD74-ROS1* fusion had a significantly longer progression-free survival (PFS) (17.63 vs. 12.63 months, *p* = 0.048) and overall survival (44.50 vs. 24.33 months, *p* = 0.036) (8). A retrospective study investigated the influence of concomitant mutations on crizotinib efficacy among 19 NSCLC patients with *ROS1* fusion. The result suggested that patients with exclusive *ROS1* fusion had a longer PFS than those with concomitant mutations (15.5 vs. 8.5 months, *p* = 0.0213) (10). In addition, the clinical characteristics related to crizotinib efficacy in patients with *ROS1*-rearranged NSCLC were analyzed, and >2 baseline metastatic organ involvement was identified as the only independent prognostic factor of PFS (hazard ratio, 4.762; 95% confidence interval, 1.515–14.961; *p* = 0.008) (11). The report of worse clinical outcomes in *ALK*-rearranged NSCLC patients whose tumors harbor nonreciprocal/reciprocal *ALK* translocation, which leads to another gene fused to *ALK* gene, reminded us to explore whether complex *ROS1* fusions influence the response of patients to crizotinib. In this study, we describe a NSCLC patient with double *ROS1* fusions who showed obvious but unsustainable response to crizotinib.

## Case Report

**Figure 1:**
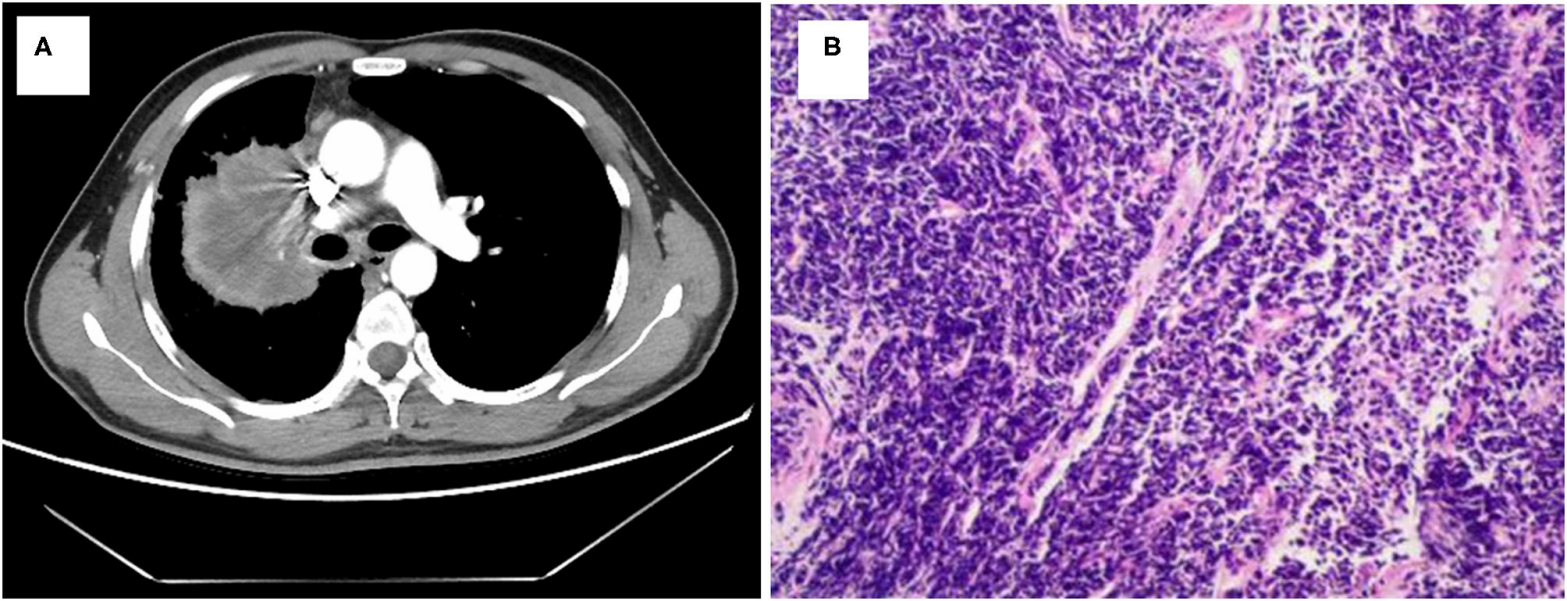
Lung adenocarcinoma shown by radiologic and pathologic examinations. **(A)** Chest CT scan reveals a mass in right lung. **(B)** Hematoxylin and eosin staining shows a low differentiation adenocarcinoma (HE × 100).

**Figure 2:**
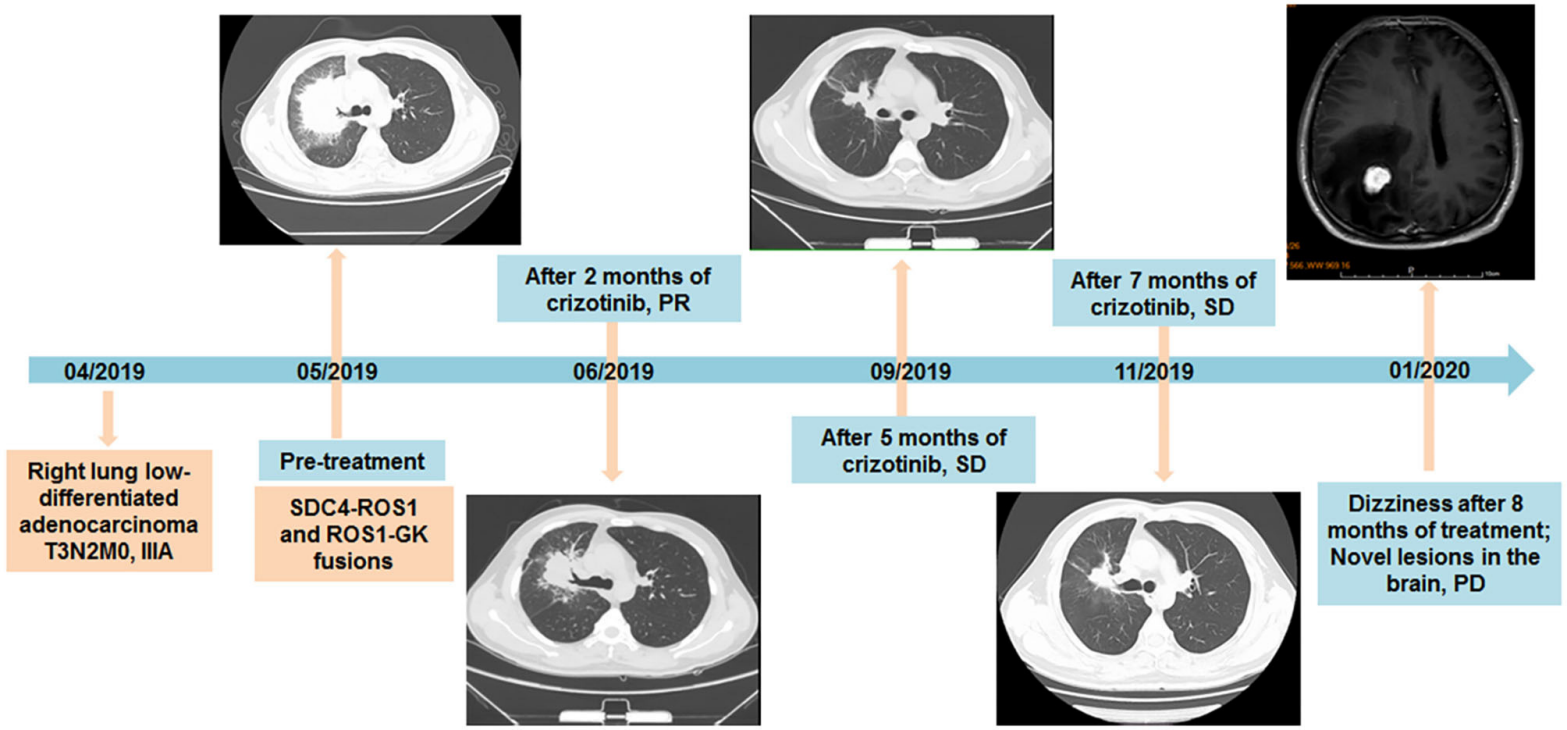
Timeline of diagnosis and treatment of the patient, with CT and MRI scans of lesions during the treatment of crizotinib additionally provided.

A 40-year-old smoking man presented to our hospital complaining of a 5-month history of aggravating and irritating dry cough in April 2019. In 1999, he underwent surgery for ulna fracture. He denied other prior disease and family history. Percussion of right upper lung showed solid sound, and auscultation suggested that the right lung breath sound was weak. The chest computed tomography (CT) showed a mass in the right lung (7.6 cm × 5.8 cm) with multiple enlarged lymph nodes in mediastinum (Figures 1A, 2). Magnetic resonance imaging (MRI) did not find any significant space-occupying lesions in the brain. Percutaneous lung biopsy was performed, and atypical cells were observed (Figure 1B). Immunohistochemical staining showed positive expression for thyroid transcription factor-1 (TTF-1), cytokeratin (CK) 8/18, and CK7, as well as negative expression for CK5/6, novel aspartic proteinase A (Napsin A), and P40. Pathological findings of the lung biopsy established the diagnosis of lung low-differentiated adenocarcinoma. Finally, his disease was diagnosed as right lung adenocarcinoma (T3N2M0, IIIA).

**Figure 3:**
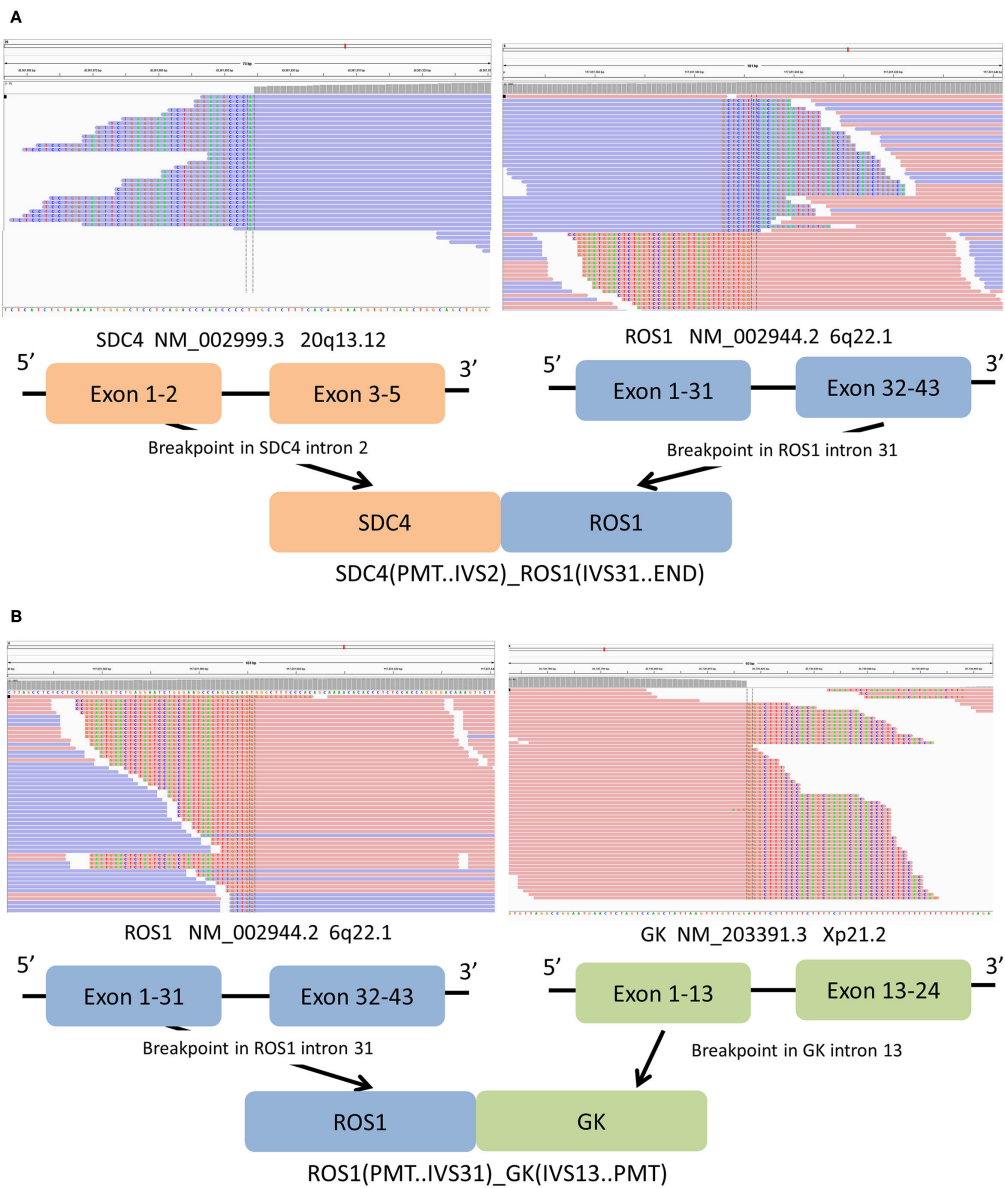
**(A)** Sequencing reads of SDC4 and *ROS1* are shown by the Integrative Genomics Viewer, and Schematic representation of the SDC4- *ROS1* fusion protein domain structure. **(B)** The Integrative Genomics Viewer snapshot of *ROS1*-GK fusion. Schematic representation of the *ROS1*-GK fusion protein domain structure. Orange, SDC4; blue, *ROS1*; blue, *ROS1*; cyan, ADGRG6.

The biopsy tissue was sent for next-generation sequencing using a commercially customized pan-cancer 1,021-gene panel that was described previously (12). A total of five somatic mutations were detected (Table 1), including SDC4-*ROS1*(EX2:EX32) and *ROS1*-GK (EX31:EX13) double *ROS1* rearrangements with mutant allele frequency of 7.0 and 7.8%, respectively (Figure 3). The other three mutations occurred in *NF1, ERBB4*, and *EPAS1* genes, respectively, without corresponding targeted agents indicated.

**Table 1:** Gene mutation results of tissue before crizotinib.

**Gene**	**c.HGVS**	**p.HGVS**	**Functional** **region**	**Allele** **frequency**
*NF1*	c.7324C>T	p.L2442F	EX49	7.6%
*ERBB4*	c.1976G>T	p.G659V	EX17	5.4%
*EPAS1*	c.469A>G	p.K157E	EX5	4.6%
*GK-ROS1*	NA	Fusion	EX13:EX31	7.8%
*SDC4-ROS1*	NA	Fusion	EX2:EX32	7.0%

*c.HGVS, description of coding DNA (c.) variants by human genome variation society (HGVS); p.HGVS, description of protein (p.) variants by HGVS*.

Considering the existence of crizotinib-sensitive *SDC4-ROS1* fusion, the patient was treated with crizotinib 250 mg orally twice daily as first-line treatment since May 2019. After 2 months of treatment, the cough significantly improved, and a follow-up CT suggested a partial response in the lung lesions (4.2 cm × 3.3 cm) according to the Response Evaluation Criteria In Solid Tumors v1.1. The follow-up CT examinations at 5 and 7 months after treatment showed that the lung lesion was stable compared with previous examinations (Figure 2). In February 2020, the patient was admitted to the hospital for 1 month of progressively aggravated headache, dizziness, vomiting, and nausea. The MRI revealed an iso-signal shadow with a diameter of 0.8 cm and 2.1 cm in the right frontal lobe and parietal lobe, respectively, suggesting metastatic lesions in the brain (Figure 2). Then, the patient was treated with gamma knife to brain lesions in March 2020. After radiotherapy, the headache of the patient was basically relieved, with occasional dizziness and no severe nausea and vomiting. Therefore, no further systemic treatment was given. In May 2020, the patient reported slight fatigue and no new symptoms and other obvious discomfort. Chest CT was performed, and no significant change was found in the lung lesions. Moreover, no significant abnormalities were found during the brain MRI reexamination. In May 2020, the patient began the treatment of traditional Chinese medicine by himself. During the follow-up inquiry in July 2020, the patient complained of anorexia, occasional nausea, and no other discomfort symptoms.

## Discussion

This study firstly described the detection of double *ROS1* rearrangements (*SDC4*-*ROS1* and *ROS1*-*GK*) in a smoking lung adenocarcinoma patient who showed the obvious but unsustainable response to crizotinib treatment. After 8 months of treatment, the patient developed symptoms of dizziness, and novel brain lesions were found. *ROS1* is one of 58 human receptor tyrosine kinases and the only member of its own subfamily (13). In this patient, exons 32–43 of *ROS1* were fused to exons 1–2 of *SDC4*. The fusion product retained the entire *ROS1* tyrosine kinase domain. *SDC4* is a common fusion partner of *ROS1*, constituting for 13% of *ROS1* fusion in Chinese patients, and intron 2 for SDC4 and intron 31 for ROS1 are common breakpoint point locations (14). Patients with *SDC4-ROS1* rearrangement were enrolled into the clinical study to access the efficacy of crizotinib in NSCLC patients with *ROS1* fusion. The median PFS for all *ROS1*-positive patients, non-*CD74-ROS1* group and *CD74-ROS1* group, was 12.63 months, 17.63 and 12.63 months, respectively (8). The 5′ *ROS1* fusion was *ROS1*-*GK*, which is a novel *ROS1* fusion. *GK* gene is located at chromosome Xp21.2 and includes 24 exons. *GK* gene is a protein coding gene belonging to the FGGY kinase family, which is a key enzyme in the regulation of glycerol uptake and metabolism (15). The expression of *GK* gene in human lung epithelial cells and NSCLC cells has been reported (16). In this novel *ROS1*-*GK* fusion, the fusion breakpoint falls into intron 31 of *ROS1*; the 5′ *ROS1* region containing several fibronectin type III regions and the 5′ *GK* region were both retained in the fusion product. The *ROS1-GK* fusion itself was speculated to be nonfunctional due to the absence of ROS1 tyrosine kinase domain. Given the prominent efficacy of crizotinib in *ROS1*-rearranged NSCLC patients, with the median PFS ranging from 15.9 to 19.3 months in different clinical studies (17, 18), the relatively shorter PFS in our case prompted us to explore potential negative factors influencing crizotinib efficacy.

NSCLC patients with *CD74-ROS1* fusion, concomitant mutations, or >2 metastatic organs have been confirmed to have a relatively inferior response to crizotinib treatment in previous studies (8–11). In this case, the patient was initially diagnosed as stage IIIA lung adenocarcinoma. Although three concomitant mutations were detected simultaneously, these mutations have not been reported to be oncogenic or influence the efficacy of crizotinib. Therefore, known negative factors could not explain the inferior survival in our patient. We noticed that several studies reported the identification of double *ALK* fusions in one patient, and high incidence of brain metastases and inferior efficacy of crizotinib treatment in this special population (19–21). *ROS1* shares extensive amino acid homology with *ALK*, especially in the kinase domain (22), and is phylogenetically related to *ALK* (23). Therefore, we speculated that *GK-ROS1* fusion in our case might contribute to the unsustainable response to crizotinib. *GK, ROS1*, and *SDC4* are located on chromosome X, 6, and 20, respectively. Double *ROS1* fusions identified in our patient suggested the more complex chromosomal abnormality, which might be associated with poor prognosis. Further i*n vitro* or *in vivo* researches may provide a stronger evidence supporting that the patient harboring double *ROS1* translocations (*GK-ROS1* and *SDC4-ROS1*) was sensitive to crizotinib treatment. Because of the low incidence rate of *ROS1* fusion in NSCLC, only one case was reported in our study. Large-scale investigations on the efficacy of ROS1 inhibitors in patients with complex *ROS1* fusions should be conducted in the future.

## Conclusion

In summary, the novel double *ROS1* rearrangements were firstly identified in a smoking, lung adenocarcinoma patient, and he responded to crizotinib in a relatively short time in first-line treatment. The shorter PFS may be associated with the presence of double *ROS1* rearrangements. Besides classical *ROS1* fusion, the identification of other concomitant *ROS1* fusions using next-generation sequencing might predict the survival outcomes of crizotinib treatment.

## Data Availability Statement

The original contributions presented in the study are included in the article/supplementary material, further inquiries can be directed to the corresponding author/s.

## Ethics Statement

The studies involving human participants were reviewed and approved by General Hospital of Northern Theater Command. The patients/participants provided their written informed consent to participate in this study. Written informed consent was obtained from the individual(s) for the publication of any potentially identifiable images or data included in this article.

## Author Contributions

LX, XC, and HH: conceptualization and design, writing, original draft, and writing review and editing. YL, XY, DG, MY, and MZ: data collection and analysis, and writing review and editing. RC and JW: conceptualization and design, and writing review and editing. ZZ: conceptualization, supervision, and writing review and editing. All author have reviewed and approved the submitted version.

## Funding

This work was supported by Wu Jiping Medical Foundation project [320.6750.16228].

## Conflict of Interest

The authors declare that the research was conducted in the absence of any commercial or financial relationships that could be construed as a potential conflict of interest.

## Publisher's Note

All claims expressed in this article are solely those of the authors and do not necessarily represent those of their affiliated organizations, or those of the publisher, the editors and the reviewers. Any product that may be evaluated in this article, or claim that may be made by its manufacturer, is not guaranteed or endorsed by the publisher.

## References

[1] Rotow J Bivona TG. Understanding and targeting resistance mechanisms in NSCLC. Nat Rev Cancer. (2017) 17:637–58. 10.1038/nrc.2017.84 29068003

[2] Ou SI Zhu VW. CNS metastasis in ROS1+ NSCLC: an urgent call to action, to understand, and to overcome. Lung Cancer. (2019) 130:201–7. 10.1016/j.lungcan.2019.02.025 30885345

[3] Collisson EA Taylor BS Campbell J Brooks AN Berger AH Chmielecki J. Comprehensive molecular profiling of lung adenocarcinoma. Nature. (2014) 511:543–50. 10.1038/nature13385 25079552 PMC4231481

[4] Facchinetti F Loriot Y Kuo MS Mahjoubi L Lacroix L Planchard D. Crizotinib-resistant ROS1 mutations reveal a predictive kinase inhibitor sensitivity model for ROS1- and ALK-rearranged lung cancers. Clin Cancer Res. (2016) 22:5983–91. 10.1158/1078-0432.CCR-16-0917 27401242

[5] Park S Ahn BC Lim SW Sun JM Kim HR Hong MH. Characteristics and outcome of ROS1-positive non-small cell lung cancer patients in routine clinical practice. J Thorac Oncol. (2018) 13:1373–82. 10.1016/j.jtho.2018.05.026 29883837

[6] Zhu VW Upadhyay D Schrock AB Gowen K Ali SM Ou SH. TPD52L1-ROS1, a new ROS1 fusion variant in lung adenosquamous cell carcinoma identified by comprehensive genomic profiling. Lung Cancer. (2016) 97:48–50. 10.1016/j.lungcan.2016.04.013 27237027

[7] Xu S Wang W Xu C Li X Ye J Zhu Y. ROS1-ADGRG6: a case report of a novel ROS1 oncogenic fusion variant in lung adenocarcinoma and the response to crizotinib. BMC Cancer. (2019) 19:769. 10.1186/s12885-019-5948-y 31382924 PMC6683537

[8] Li Z Shen L Ding D Huang J Zhang J Chen Z. Efficacy of crizotinib among different types of ROS1 fusion partners in patients with ROS1-rearranged non-small cell lung cancer. J Thorac Oncol. (2018) 13:987–95. 10.1016/j.jtho.2018.04.016 29704675

[9] Landi L Chiari R Tiseo M D'Incà F Dazzi C Chella A. Crizotinib in MET-deregulated or ROS1-rearranged pretreated non-small cell lung cancer (METROS): a phase II, prospective, multicenter, two-arms trial. Clin Cancer Res. (2019) 25:7312–9. 10.1158/1078-0432.CCR-19-0994 31416808

[10] Zeng L Li Y Xiao L Xiong Y Liu L Jiang W. Crizotinib presented with promising efficacy but for concomitant mutation in next-generation sequencing-identified ROS1-rearranged non-small-cell lung cancer. Onco Targets Ther. (2018) 11:6937–45. 10.2147/OTT.S176273 30410351 PMC6199224

[11] Zheng J Cao H Li Y Rao C Zhang T Luo J. Effectiveness and prognostic factors of first-line crizotinib treatment in patients with ROS1-rearranged non-small cell lung cancer: a multicenter retrospective study. Lung Cancer. (2020) 147:130–6. 10.1016/j.lungcan.2020.07.016 32702569

[12] Lin G Li C Li PS Fang WZ Xu HP Gong YH. Genomic origin and EGFR-TKI treatments of pulmonary adenosquamous carcinoma. Ann Oncol. (2020) 31:517–24. 10.1016/j.annonc.2020.01.014 32151507

[13] Blume-Jensen P Hunter T. Oncogenic kinase signalling. Nature. (2001) 411:355–65. 10.1038/35077225 11357143

[14] Cui M Han Y Li P Zhang J Ou Q Tong X. Molecular and clinicopathological characteristics of ROS1-rearranged non-small-cell lung cancers identified by next-generation sequencing. Mol Oncol. (2020) 14:2787–95. 10.1002/1878-0261.12789 32871626 PMC7607175

[15] Torres-García D Pérez-Torres A Manoutcharian K Orbe U Servín-Blanco R Fragoso G. GK-1 peptide reduces tumor growth, decreases metastatic burden, and increases survival in a murine breast cancer model. Vaccine. (2017) 35:5653–61. 10.1016/j.vaccine.2017.08.060 28890195

[16] Fagerberg L Hallström BM Oksvold P Kampf C Djureinovic D Odeberg J. Analysis of the human tissue-specific expression by genome-wide integration of transcriptomics and antibody-based proteomics. Mol Cell Proteomics. (2014) 13:397–406. 10.1074/mcp.M113.035600 24309898 PMC3916642

[17] Wu YL Yang JC Kim DW Lu S Zhou J Seto T. Phase II study of crizotinib in east Asian patients with ROS1-positive advanced non-small-cell lung cancer. J Clin Oncol. (2018) 36:1405–11. 10.1200/JCO.2017.75.5587 29596029

[18] Shaw AT Riely GJ Bang YJ Kim DW Camidge DR Solomon BJ. Crizotinib in ROS1-rearranged advanced non-small-cell lung cancer (NSCLC): updated results, including overall survival, from PROFILE 1001. Ann Oncol. (2019) 30:1121–6. 10.1093/annonc/mdz131 30980071 PMC6637370

[19] Luo J Gu D Lu H Liu S Kong J. Coexistence of a Novel PRKCB-ALK, EML4-ALK double-fusion in a lung adenocarcinoma patient and response to crizotinib. J Thorac Oncol. (2019) 14:e266–e8. 10.1016/j.jtho.2019.07.021 31757376

[20] Su Y Long X Song Y Chen P Li S Yang H. Distribution of ALK fusion variants and correlation with clinical outcomes in chinese patients with non-small cell lung cancer treated with crizotinib. Target Oncol. (2019) 14:159–68. 10.1007/s11523-019-00631-x 30895431

[21] Zhang Y Zeng L Zhou C Li Y Wu L Xia C. Detection of nonreciprocal/reciprocal ALK translocation as poor predictive marker in patients with first-line crizotinib-treated ALK-rearranged NSCLC. J Thorac Oncol. (2020) 15:1027–36. 10.1016/j.jtho.2020.02.007 32112982

[22] Ou SH Tan J Yen Y Soo RA. ROS1 as a ‘druggable’ receptor tyrosine kinase: lessons learned from inhibiting the ALK pathway. Expert Rev Anticancer Ther. (2012) 12:447–56. 10.1586/era.12.17 22500682

[23] Robinson DR Wu YM Lin SF. The protein tyrosine kinase family of the human genome. Oncogene. (2000) 19:5548–57. 10.1038/sj.onc.1203957 11114734

